# Experience of a Tertiary Service in the Treatment of Women with Cervical Pregnancy

**DOI:** 10.1055/s-0042-1757954

**Published:** 2022-12-29

**Authors:** Karen Hiromi Mori, Bárbara Virgínia Tavares, Daniela Angerame Yela, Luis Francisco Cintra Baccaro, Cassia Raquel Teatin Juliato

**Affiliations:** 1 Universidade Estadual de Campinas, Campinas, SP, Brazil

**Keywords:** cervical pregnancy, ectopic pregnancy, methotrexate, surgery, treatment, gravidez cervical, gravidez ectópica, metotrexato, cirurgia, tratamento

## Abstract

**Objective** Cervical pregnancy is challenging for the medical community, as it is potentially fatal. The treatment can be medical or surgical; however, there are no protocols that establish the best option for each case. The objective of the present study was to describe the cases of cervical pregnancy admitted to a tertiary university hospital over a period of 18 years.

**Methods** A retrospective study based on a review of the medical records of all cervical pregnancies admitted to the Women's Hospital at Universidade Estadual de Campinas, Southeastern Brazil, from 2000 to 2018.

**Results** We identified 13 cases of cervical pregnancy out of a total of 673 ectopic pregnancies; only 1 case was initially treated with surgery because of hemodynamic instability. Of the 12 cases treated conservatively, 7 were treated with single-dose intramuscular methotrexate, 1, with intravenous and intramuscular methotrexate, 1, with intravenous methotrexate, 1, with 2 doses of intramuscular methotrexate, and 2, with intra-amniotic methotrexate. Of these cases, one had a therapeutic failure that required a hysterectomy. Two women received blood transfusions. Four women required cervical tamponade with a Foley catheter balloon for hemostasis. There was no fatal outcome.

**Conclusion** Cervical pregnancy is a rare and challenging condition from diagnosis to treatment. Conservative treatment was the primary method of therapy used, with satisfactory results. In cases of increased bleeding, cervical curettage was the initial treatment, and it was associated with the use of a cervical balloon for hemostasis.

## Resumo

**Objetivo** A gravidez ectópica cervical é um desafio para a comunidade médica, pois pode ser fatal. O tratamento pode ser clínico ou cirúrgico, mas não existem protocolos que estabeleçam a melhor opção para cada caso. O objetivo deste estudo foi descrever os casos de gravidez ectópica cervical internados em um hospital universitário terciário durante 18 anos.

**Métodos** Estudo retrospectivo com revisão de prontuários de todas as gestações ectópicas cervicais internadas no Hospital da Mulher da Universidade Estadual de Campinas de 2000 a 2018.

**Resultados** Foram identificados treze casos de gestação ectópica cervical em um total de 673 gestações ectópicas; apenas 1 caso foi inicialmente tratado com cirurgia por causa de instabilidade hemodinâmica. Dos 12 casos tratados conservadoramente, 7 foram tratados com metotrexato por via intramuscular em dose única, 1, com metotrexato pelas vias intravenosa e intramuscular, 1, com metotrexato por via intravenosa, 1, com 2 doses de metotrexato por via intramuscular, e 2, com metotrexato por via intra-amniótica. Desses casos, um apresentou falha terapêutica, e realizou-se uma histerectomia. Duas mulheres receberam transfusões de sangue. Quatro mulheres necessitaram de tamponamento cervical com cateter balão de Foley para hemostasia. Não houve casos fatais.

**Conclusão** A gravidez cervical é uma condição rara e desafiadora desde o diagnóstico até o tratamento. O tratamento conservador foi o principal método terapêutico utilizado, com resultados satisfatórios. Nos casos de sangramento aumentado, a curetagem cervical foi o tratamento inicial, e foi associada ao uso de balão cervical para hemostasia.

## Introduction

Cervical ectopic pregnancy is defined as the implantation of the embryo into the endocervical canal. The pathophysiology of the condition is not completely understood. Several studies 1 have suggested that surgical procedures such as dilatation and curettage, in addition to cesarean sections, are associated with a higher occurrence of this condition. Assisted reproduction procedures have also been associated with a higher frequency of cervical ectopic pregnancies. 2 3 4 5 6 The condition is rare, with an estimated incidence of 0.0001%, that is, 1 in every 10 thousand women will have a cervical pregnancy. 3

The most common symptoms of cervical pregnancy are vaginal bleeding and pain. However, the trophoblastic invasion that occurs in the epithelium and endocervical fibromuscular stroma can lead to rupture of the local vessels, resulting in profuse hemorrhage. If a cervical pregnancy is not promptly treated, there is a high risk of severe hemorrhage, which can lead to maternal morbidity and even death. 7 From 2000 to 2019, it is estimated that 1.9% of maternal deaths in the state of São Paulo (the most developed region in Brazil) occurred due to complications of cervical ectopic pregnancy. 8 Thus, the appropriate treatment for ectopic pregnancy is a challenge for the medical community.

As it is a rare disease, there is no well-established protocol for its management. 9 Previously, cervical pregnancy was treated surgically, primarily by hysterectomy due to the high risk of severe hemorrhage. In recent years, most reports 10 11 have demonstrated the high efficacy and safety of the conservative medical treatment for cervical pregnancy with systemic or local methotrexate (MTX), uterine artery embolization, or dilation and curettage (D&C) with Foley catheter balloon tamponade. However, to date, no consensus has been reached among clinicians regarding the standard care for cervical ectopic pregnancy. The aim of the present study was to describe our experience with 13 cases of cervical pregnancy at a tertiary teaching hospital in Southeastern Brazil throughout 18 years.

## Methods

The present is a retrospective study that involved the evaluation of medical records of women with cervical pregnancies admitted to a tertiary hospital (Centro de Atenção Integral à Saúde da Mulher [CAISM], the Women's Hospital at Universidade Estadual de Campinas) from 2000 to 2018. We identified the cases in the hospital records based on the International Classification of Diseases. The study was approved by the Research Ethics Committee under a Certificate of Presentation for Ethical Appreciation (number 53019116.6.0000.5404). The research followed the principles of the Declaration of Helsinki.

A diagnosis of cervical ectopic pregnancy was made through transvaginal ultrasound and involved the identification of a gestational sac in the uterine cervix and the absence of intrauterine pregnancy. In cervical ectopic pregnancies with an embryo, the presence of cardiac activity in the embryo was evaluated, in addition to other ultrasound descriptors such as the shape of the uterus (if it was described as having an hourglass shape with a ballooned cervical canal). 12

We obtained the clinical and obstetric history by reviewing the medical records. The gestational age was calculated based on the date of the last menstrual period or was estimated by ultrasound (US) when necessary. The following factors associated with cervical ectopic pregnancy were evaluated: gestational age, ultrasound diagnosis, the presence of fetal cardiac activity, treatment methods, and success rate. The treatment (either medical or surgical) was considered successful if a negative beta-human chorionic gonadotropin (β-hCG) result was obtained.

The quantitative variables were presented as means and standard deviations. The categorical variables were presented as absolute numbers and relative frequencies.

## Results

**Fig. 1:**
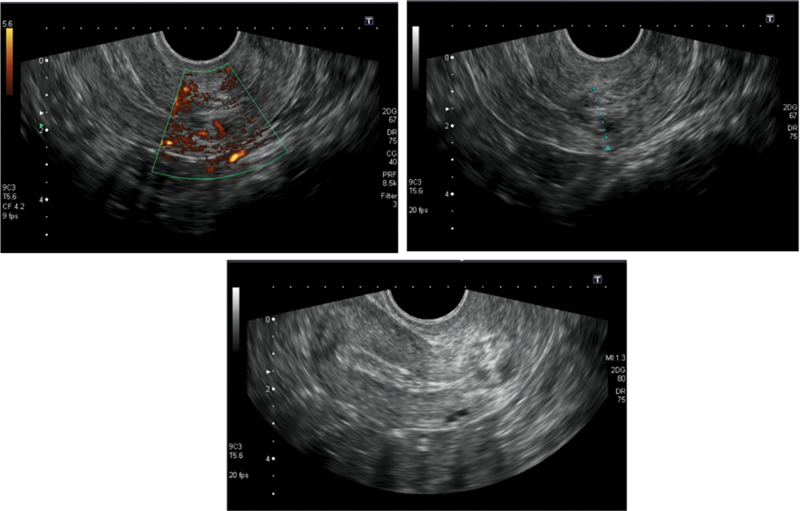
Ultrasonography aspects of cervical ectopic pregnancy.

We identified 673 women with ectopic pregnancies, and 13 (1.9%) presented ultrasonography criteria for a cervical ectopic pregnancy ( Fig. 1 ).

The mean age of the women with a cervical ectopic pregnancy was of 31.8 (±5.9) years, and the number of previous pregnancies was of 2.1 (±1.4). Approximately half of the women (7/13; 53.8%) had a history of cesarean section, with an average of 0.8 (±0.9). A history of uterine curettage was present in 2 of them, with an average of 0.3 (±0.8). None of the women used an intrauterine device as a contraceptive method or reported that they smoked ( Chart 1 ).

**Chart 1:** Characteristics and treatment of the cases of cervical pregnancy

N ^O^	Age (years)	Parity index	GA	Initial diagnosis	Ultrasonography findings	β-hCG (IU/l)	Initial treatment	Additional treatment
**1**	39	G2C1	6 + 2	Anembryonic pregnancy	4-cm cervical gestational sac	129,57	Methotrexate IM	None
**2**	26	G6C2A3	6 + 5	Cervical ectopic pregnancy	Hematometra 79 × 47 × 62 mm, cervical gestation, and invasion of decidua into the myometrium, FHB present	88,878	Cervical curettage, vacuum aspiration, Foley catheter balloon	None
**3**	30	G3C1A1	10 + 5	Cervical ectopic pregnancy	Isthmic pregnancy, CRL 38 mm, FHB present	75,810	AF aspiration, intracardiac injection of xylocaine, and intra-amniotic injection of methotrexate 50 mg	None
**4**	32	G6P5C3	9 + 5	Cervical ectopic pregnancy in abortion	Cervical ectopic pregnancy in abortion: heterogeneous oval image, 5.7 × 8.4 mm	4,585	Methotrexate EV and methotrexate IM	None
**5**	28	G4P2A1	8 + 0	Embryonic death in the cervical region	Gestational sac 25 × 19 × 14 mm, with 12-mm embryo in the cervical region, without FHB	3,678	Methotrexate IM	None
**6**	35	G3P1A1	9 + 3	Cervical ectopic pregnancy	28-mm gestational sac with an embryo, 11 mm distant from the IO, FHB present	≥ 10,00	AF aspiration, intracardiac injection of xylocaine and intra-amniotic injection of methotrexate 87.5 mg	None
**7**	31	G3C2	6 + 1	Cervical ectopic pregnancy	Cervical ectopic pregnancy of GA 6 + 2, with live embryo	≥ 10,000	Methotrexate IM	Cervical curettage
**8**	25	G3C1A1	10 + 2	Ectopic pregnancy in Cesarean section scar	Cervical region just below cesarean section scar – an image of 23 × 27 × 33 mm may correspond to cervical ectopic pregnancy	642	Methotrexate IM	None
**9**	21	G3P1A1	9 + 0	Incomplete abortion in the cervical canal	Heterogeneous nodular formation in the cervical canal with extension to the serosa measuring 42 × 38 × 35mm, with gestational sac without embryo	6,598	Methotrexate IM	Curettage and Foley catheter balloon
**10**	37	G3P2C1	9 + 0	Cervical ectopic pregnancy	Cervical ectopic pregnancy with the presence of the gestational sac only	10,185	Methotrexate IM	None
**11**	44	G1P0	7 + 1	Cervical ectopic pregnancy	Pregnancy in the cervical-uterine body transition, with embryo and FHB present	78,839	Methotrexate EV	Curettage, Foley catheter balloon, hysterectomy
**12**	33	G2A1	5 + 6	Cervical ectopic pregnancy	Anechoic image in cervical region 21 × 13 × 11mm, diameter: 15mm, 2.9-mm CRL embryo, FHB present	7,683	Methotrexate IM	Methotrexate IM
**13**	33	G1	6 + 3	Cervical embryonic death	Gestational sac 14 × 6 × 11 mm, with implantation on the cervix, with no sign of rupture, with Doppler flux, presence of an embryo	6,548	Methotrexate IM	Cervical Foley catheter

Abbreviations: A, abortion; AF, amniotic fluid; C, cesarean delivery; CRL, crown-rump length; EV, endovenous; FHB, fetal heartbeat; G, gravity; GA, gestational age; IM, intramuscular; IO, internal os; P, deliveries; β-hCG, beta-human chorionic gonadotropin.

**Fig. 2:**
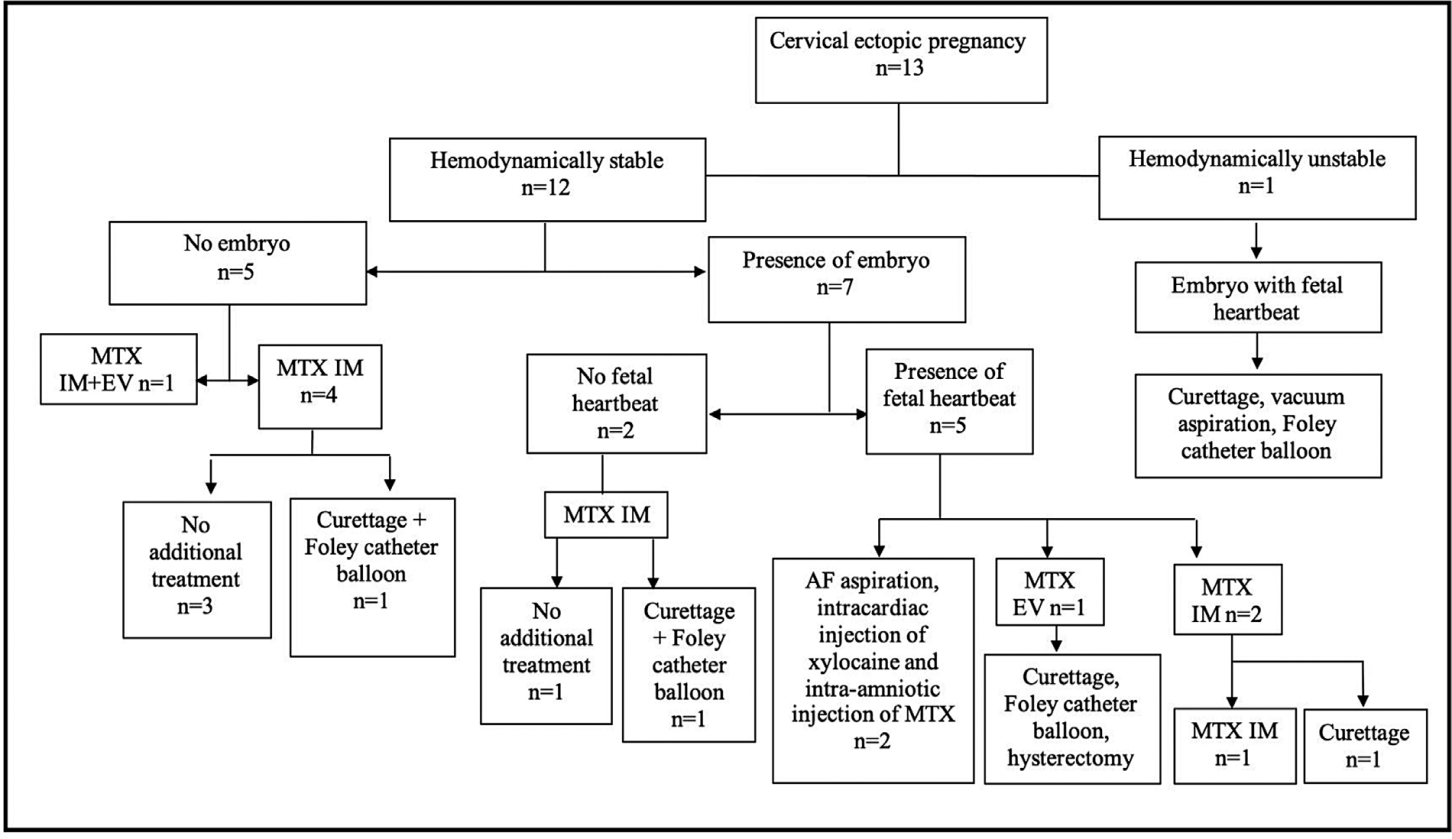
Flowchart of the treatment for cervical ectopic pregnancy. Abbreviations: AF, amniotic fluid; EV, endovenous; IM, intramuscular; MTX, methotrexate; n, number.

Among the 13 identified cases, the average gestational age was of 8.1 (±1.7) weeks. Of these women, 69.2% had vaginal bleeding on admission, and 2 (15.3%) had profuse bleeding. On admission, 4 women (30.7%) complained of pain in the lower abdomen. The mean β-hCG was of 2,3351.96 (±SD) IU/L. In the ultrasound evaluation, the embryos of 8 out of the 13 women (61.5%) could be visualized and the embryos of 6 of these had visible heartbeats. The medical management involved systemic or intra-amniotic MTX injection after an evaluation of the blood tests regarding liver and renal function and a complete blood count. The baseline serum β-hCG was recorded. The MTX injection was administered as follows: an intramuscular dose of 50 mg/m ^2^ , an intravenous dose of 1mg/kg, or an intra-amniotic dose of 1 mg/kg. The serum levels of β-hCG were assessed on days 4 and 7 after the injection, and a ≥ 15% drop between these days was considered a response to the medical treatment. Subsequently, the levels of β-hCG were monitored weekly until they dropped below 5 mIU/mL, when they were considered negative. Regarding the treatment, a flowchart of the identified cases is presented in Fig. 1 . Of the 13 cases, 1 required immediate surgical treatment after admission due to heavy bleeding and hemodynamic instability. In this case, the presence of an embryo with a fetal heartbeat was identified during the ultrasound. The patient was treated with cervical canal curettage, vacuum aspiration, and insertion of a Foley catheter balloon with 30 mL of water for hemostasis. The patient also received a blood transfusion. The 12 remaining hemodynamically stable cases were divided into 2 groups based on the ultrasound results: 7 women with an embryo and 5 women without an embryo ( Fig. 2 ).

### Cervical Pregnancy without an Embryo during an Ultrasound Evaluation

Among the 5 women without embryos, 3 were successfully treated with a single administration of intramuscular MTX (50 mg/m ^2^ ). One woman received a double dose of MTX, one received an intramuscular dose, and another received an intravenous dose. The remaining patient was treated with a single intramuscular dose of MTX and also required complementary treatment with uterine curettage and homeostasis with a Foley catheter balloon. The patient returned eleven days after the MTX injection with severe abdominal pain and excessive vaginal bleeding. The bleeding was maintained despite the curettage, so the Foley balloon catheter was positioned with 60 mL of water. The balloon was removed after 24 hours, with no further complications. The patient required a blood transfusion after the curettage.

### Cervical Pregnancy with an Embryo but without a Heartbeat during an Ultrasound Evaluation

The two women who had embryos without a heartbeat during the ultrasound evaluation were treated with a single dose of intramuscular MTX. In 1 of the women, the initial β-hCG level was of 3,678 UI/L. After four days of MTX administration, the patient developed vaginal bleeding, and the cervical pregnancy was aborted. The control ultrasound showed no evidence of ectopic pregnancy on the cervix. The β-hCG level on day 9 after the administration of MTX decreased to 10 UI/L. In the other patient, the initial β-hCG level was of 6,548 UI/L. On day 6 after the MTX injection, the woman presented with vaginal bleeding and the gestational sac was adhered to the cervix. An attempt to remove the sac was unsuccessful due to its strong adherence to the cervix. A Foley balloon catheter with 30 mL of water was the option of additional treatment. It was positioned, guided by an ultrasound, and was removed after 48 hours with no complications. The β-hCG level decreased from 30,188 UI/L on day 4 to 4,819 UI/L on day 7.

### Cervical Pregnancy with an Embryo with a Heartbeat during an Ultrasound Evaluation

There were 5 cases of embryos with a heartbeat, and three different treatment protocols were used. Two of the cases were treated with an intramuscular MTX injection (50 mg/m ^2^ ). In these two cases, due to elevated serum levels of β-hCG, one woman underwent uterine curettage, and the other received a second dose of intramuscular MTX (50 mg/m ^2^ ). In this latter case, an ultrasound performed on day 4 after the MTX injection showed evidence of an embryo with a heartbeat. One of the 5 cases received a dose of intravenous MTX (1 mg/kg); however, due to therapeutic failure and an elevated β-hCG level, the patient required additional treatment and underwent curettage and hemostasis with a Foley catheter balloon. The condition progressed, and the patient suffered excessive bleeding; thus, a hysterectomy was performed. This was the only case that required a hysterectomy after failed hemostasis with a Foley catheter balloon. The other two cases required aspiration of 30 mL of amniotic fluid and intracardiac injection of xylocaine and intra-amniotic injection of MTX. One of the patients received 50 mg of intra-amniotic MTX after the heartbeat of the embryo stopped. The other case received 87.5 mg of intra-amniotic MTX after the heartbeat of the embryo stopped.

## Discussion

The present study described 18 years of experience concerning the treatment of cervical ectopic pregnancy, and positive results were observed regarding the conservative treatment. However, after the medical treatment, there was a considerable requirement for complementary treatment.

The results of the present study were positive because only one woman required a hysterectomy for a cervical pregnancy. A separate study 13 demonstrated that 70% of cervical pregnancies required a hysterectomy due to massive blood loss. Most of the women included in this study were hemodynamically stable. In these cases, medical treatment with MTX could be considered as a first-line treatment. Isolated curettage is associated with a 40% risk of requiring a hysterectomy. 14 Treatment with MTX was initially conducted in 1983 15 and, at present, the literature shows that the risk of bleeding is of 11% for drug treatment and of 3% in the case of a hysterectomy. 11 Recent studies 16 17 show that drug treatment can prevent a hysterectomy in up to 91% of cases. The conservative treatments include drug treatment (local or systemic treatments, or treatments with intracervical MTX, potassium chloride, vasopressin), and surgical procedures (local aspiration, curettage, cerclage with cervical tamponade), and the radical treatments include cervical amputation and hysterectomy. 11

In the present study, the treatment with MTX was initially only successful in 4 out of 8 women with a cervical pregnancy. However, an intramuscular single dose of this medication was used, and, in certain cases, it was used heterogeneously with the application of intravenous doses. Evidence of the use of MTX for the treatment of cervical pregnancy is limited to case reports. 18 19 20 21 Previous studies reported cases of successful treatment of cervical ectopic pregnancies with multiple doses of MTX, suggesting that it is an appropriate therapy for hemodynamically stable women. 22 23 A separate study 24 compared the effectiveness of the treatment with a single and with multiple doses of MTX, and the authors concluded that a single dose was as effective as multiple doses. Since the condition is rare and the number of cases was limited, in the present study, we could not conclude whether the failure of the initial treatment in our case series was solely due to the MTX regimen adopted.

Although experience with cervical ectopic pregnancy is limited to conclusions concerning the criteria for medical therapy, it has been postulated that serum levels of β-hCG > 10,000 mUI/ mL, gestational age > 9 weeks, the presence of cardiac fetal activity, or a crown-rump length > 10 mm will result in unsuccessful treatment of the cervical ectopic pregnancy. 11 Similar to observations made regarding tubal pregnancy, the presence of fetal cardiac activity is a poor prognostic factor for successful MTX treatment. 25 However, in addition to this poor prognostic factor, the surgical risk of hemorrhage is higher in cervical pregnancies than in tubal pregnancies. In the present study, two women with fetal heartbeat on ultrasound were successfully treated with intra-amniotic MTX. This result corroborates those of a recent study 26 that demonstrated greater efficacy when intra-amniotic MTX was used after the systemic use failed. Previous studies 27 described the injection of potassium chloride or intracardiac ethanol in these cases, and the authors demonstrated an improvement in the effectiveness of the conservative treatment. However, the data are too limited to enable a comparison of the two regimes. 11 In the present study, there was a successful report of xylocaine injection associated with the withdrawal of amniotic fluid.

The use of curettage and hemostasis with a Foley catheter tamponade was described in other studies 28 as an alternative and safe treatment to the use of MTX. One study 29 described a series of thirteen cases successfully treated using this method, and the author emphasized that it would eliminate the maternal MTX risks. However, in the cases examined in the present study, all the women who were stabilized received either an intramuscular, intravenous, or intra-amniotic injection of MTX. Moreover, similar to other studies, 28 curettage and a Foley catheter balloon were used as additional treatments, and occasionally it was necessary to reduce the massive hemorrhage caused by the MTX at the implantation site.

The present study was conducted in a tertiary hospital, which is a reference regarding the treatment of ectopic pregnancies, as a considerable number of women with this condition have been treated there. Because cervical ectopic pregnancy is a rare condition, most of the works published in the literature are case reports. Therefore, the present was a retrospective study that included descriptions of various treatment modalities, and the results highlight the need for further studies and the establishment of protocols to assist women with cervical ectopic pregnancies.

In conclusion, from diagnosis to treatment, cervical pregnancy is a rare and challenging condition. A single intramuscular injection of MTX was the main treatment performed on women with this condition, and satisfactory results were obtained. However, there was a frequent requirement for complementary treatment due to vaginal bleeding or failure of β-hCG levels to decrease. In cases of increased bleeding, surgical treatment with curettage was the initial treatment and, in three cases, additional treatment with a cervical Foley catheter balloon for hemostasis was administered. Therefore, conservative and fertility-sparing treatment was possible in most cases. Moreover, even though cervical pregnancy has been associated with considerable morbidity, no fatal outcomes were observed during the present study.
